# Simulating atmospheric drought: Silica gel packets dehumidify mesocosm microclimates

**DOI:** 10.1002/ece3.70139

**Published:** 2024-08-21

**Authors:** S. Varghese, B. A. Aguirre, F. Isbell, A. J. Wright

**Affiliations:** ^1^ Department of Biological Sciences California State University Los Angeles Los Angeles California USA; ^2^ Department of Ecology, Evolution and Behavior University of Minnesota Minneapolis Minnesota USA; ^3^ Department of Ecology and Evolutionary Biology Cornell University Ithaca New York USA

**Keywords:** atmospheric drought, dehumidification, microclimate, relative humidity, silica gel, vapor pressure deficit

## Abstract

As global temperatures rise, droughts are becoming more frequent and severe. To predict how drought might affect plant communities, ecologists have traditionally designed drought experiments with controlled watering regimes and rainout shelters. Both treatments have proven effective for simulating soil drought. However, neither are designed to directly modify atmospheric drought. Here, we detail the efficacy of a silica gel atmospheric drought treatment in outdoor mesocosms with and without a co‐occurring soil drought treatment. At California State University, Los Angeles, we monitored relative humidity, temperature, and vapor pressure deficit every 10 min for 5 months in bare‐ground, open‐top mesocosms treated with soil drought (reduced watering) and/or atmospheric drought (silica dehumidification packets suspended 12 cm above soil). We found that silica packets dehumidified these mesocosm microclimates most effectively (−5% RH) when combined with reduced soil water, regardless of the ambient humidity levels of the surrounding air. Further, packets increased microclimate vapor pressure deficit most effectively (+0.4 kPa) when combined with reduced soil water and ambient air temperatures above 20°C. Finally, packets simulated atmospheric drought most consistently when replaced within 3 days of deployment. Our results demonstrate the use of silica packets as effective dehumidification agents in outdoor drought experiments. We emphasize that incorporating atmospheric drought in existing soil drought experiments can improve our understandings of the ecological impacts of drought.

## Graphical abstract

We detail herein the efficacy of a silica gel atmospheric drought treatment in outdoor mesocosms with and without a co‐occurring soil drought treatment. We found that silica packets dehumidified these microclimates most effectively (−5% RH) when combined with reduced soil water, regardless of the ambient humidity levels of the surrounding air. Further, packets increased microclimate vapor pressure deficit most effectively (+0.4 kPa) when combined with reduced soil water and ambient air temperatures above 20°C.

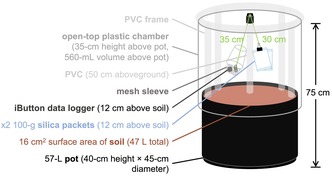

## 1 INTRODUCTION

Climate change is driving shifts in the frequency and intensity of drought worldwide (Cook et al., 2014; Grossiord et al., 2020; Hoerling et al., 2012; IPCC, 2021; Sheffield & Wood, 2008; Trenberth, 2011). In particular, warming is decreasing air relative humidity and increasing vapor pressure deficit, collectively driving higher evaporative demand (Burke & Brown, 2008; Cook et al., 2014). While drought can be defined in many ways (Table S1; Crausbay et al., 2017; Slette et al., 2019; Van Loon, 2015), the most widespread outcome of climate warming is *ecological drought*, or the combination of precipitation shortages and increased evaporative demand due to rising temperatures (IPCC, 2021). In the United States, climate models predict that summer evaporative demand (measured using vapor pressure deficit) will increase by 51% by the year 2100 (Ficklin & Novick, 2017) and has already increased by 0.05 kPa in the past century (Yuan et al., 2019).

In plant communities, drought can cause declines in species richness, increases in species extinction risk, and widespread vegetation die‐off, which can have lasting impacts on ecosystem dynamics (Allen et al., 2015; Breshears et al., 2005; McDowell et al., 2008; Tilman & El Haddi, 1992). Plants require water to perform basic metabolism. Importantly, relative water balance within a plant is the result of both water intake from the soil and water loss at the leaf surface (Schweiger et al., 2023). The later occurs via transpiration, which non‐CAM vascular land plants regulate by reducing their stomatal aperture (Lin et al., 2015; McAdam & Brodribb, 2015; Von Caemmerer & Baker, 2007). During ecological drought, plants contract stomata in response to moisture shortages in both the soil and the air, which can result in reduced productivity and decreased carbon fixation at an ecosystem scale (Fu et al., 2022; Grossiord et al., 2020; Ocheltree et al., 2014; Schönbeck et al., 2022).

Soil moisture and atmospheric demand do not always change in tandem (Fu et al., 2022; Hanks, 2012; Hillel, 2012; Novick et al., 2016). For example, while atmospheric aridity is expected to increase worldwide as a result of warming, climate models predict that changes in precipitation (e.g., “meteorological drought,” Table S1) will be more variable (Burke & Brown, 2008; Cook et al., 2014; Yuan et al., 2019). For example, in Southern California, one theory predicts a continuation of the meteorological drought conditions that have persisted for the last 20 years (Mann & Gleick, 2015), while another theory predicts *increases* in precipitation due to shifts in late‐season monsoon weather patterns (Cook & Seager, 2013). Moving forward, it will become critical to assess how ecosystems might be impacted by the independent and potentially interacting effects of moisture shortages both belowground (*soil drought*, a direct result of meteorological drought) and aboveground (*atmospheric drought*, a direct result of ecological drought; Fu et al., 2022; IPCC, 2021; Novick et al., 2016; Table S1).

Traditionally, outdoor drought experiments have manipulated soil moisture. This is typically done by restricting soil water input as a drought treatment (at mesic sites) or by increasing soil water input in comparison to already occurring drought (at arid sites) (e.g., Alster et al., 2013; Báez et al., 2013; Baldini & Vannozzi, 1999; Copeland et al., 2016; Filazzola et al., 2018; Kreyling et al., 2016). While both practices can effectively approximate soil drought, they are not designed to directly modify atmospheric drought (Aguirre et al., 2021; Kreyling et al., 2016; Rana et al., 2004; Wright & Collins, 2023; Yahdjian & Sala, 2002). Another popular approach combines soil water removals with warming treatments (e.g., Cowles et al., 2016; Mas et al., 2023; Schönbeck et al., 2022). Such studies have revealed key insights into how plant communities might respond to the combination of precipitation deficits and higher evaporative demand indirectly via increased temperatures. However, it is challenging under these conditions to separate direct temperature effects from indirect atmospheric drying effects, which plants may respond to in opposite directions (Schönbeck et al., 2022; Wright & Collins, 2023).

Combining soil drought treatments with experimental manipulations of air humidity may provide more comprehensive insights into ecological drought (Wright & Collins, 2023). For example, a study by Aguirre et al. (2021) found that grass community biomass was unaffected by soil drought when humidity was increased but was reduced by approximately 50% when soil drought occurred alongside ambient (naturally lower) air humidity. Another study conducted within this same experiment demonstrated that a focal species (*Poa secunda*) exhibited a higher root: shoot biomass ratio and lower leaf area only when soil drought was combined with naturally lower air humidity (Watson et al., 2023). Other grass species in this experiment exhibited directionally independent (and potentially interacting) growth based on whether soil drying or atmospheric drying was applied; this led to dramatic shifts in community assemblages after multiple growing seasons (Huynh et al., 2023).

Aguirre et al. (2021) proposed the use of silica gel packets as a low‐cost, low‐tech means of removing air moisture and thus maintaining atmospheric drought in pots simultaneously treated with soil drought. These authors reported a season‐wide reduction in mesocosm relative humidity (−2.5%) in pots with silica packets, though this did not translate to a corresponding season‐wide increase in vapor pressure deficit (Aguirre et al., 2021). Indeed, it is unclear how environmental conditions influence silica packet performance. For example, humid days (or times of day) might allow for stronger packet dehumidification effects (and increased vapor pressure deficit effects), while low humidity may drive weaker effects (Aguirre et al., 2021). Such effects may be particularly relevant in Mediterranean Southern California (where this experiment was set up) as this region experiences rapid diurnal shifts in hot, dry daytime conditions and cool, humid nighttime conditions.

Importantly, plants also respond to drought on a rapid timescale: reductions in leaf water potential, stem hydraulic conductance, and stomatal pore aperture have all been recorded responding to soil moisture deficits within minutes (Christmann et al., 2007; Lawson & Blatt, 2014; Saliendra et al., 1995). But it is unclear whether silica packets, as passive dehumidification apparatuses, can keep up with such rapid physiological responses to dynamic environmental cues in real time.

Finally, Aguirre et al. (2021) demonstrated that pots receiving more soil water (independent of humidity manipulations) had higher relative humidity and lower vapor pressure deficit, likely due to moisture exchange between the soil surface and the air (e.g., Zhou et al., 2019). Higher temperatures may intensify soil‐to‐air water transfer (often referred to as “land‐atmosphere feedbacks”) because soil evaporation is temperature dependent (Zhou et al., 2019). Indeed, warming has been shown to intensify soil drought at landscape scales due to increased soil surface evaporation (Dirmeyer et al., 2012; Hanks, 2012; McHugh et al., 2015; Samaniego et al., 2018). To better understand packet performance as moisture is exchanged between the soil and air (and potentially through the packets, as well), it is necessary to conduct a holistic assessment of packet responses to environmental fluctuations and feedbacks.

There are also gaps in our mechanistic understandings of how packets should be used to maintain or maximize their potential as an atmospheric drought treatment. For example, it remains unclear how long a packet can be deployed in the field before fully saturating with moisture and no longer dehumidifying the microclimate. It is also unknown whether a saturated packet left in the field might emit the moisture it previously captured. Further, a packet deployed for multiple days in a row may perform better or worse over time due to cumulative saturation effects with respect to the preceding days' temperature and humidity levels. As such, it is critical to assess how packets respond to potential interactions between the environment and how the treatment is applied, such as through replacement frequency or deployment duration.

To help address these knowledge gaps and communicate the level of detail necessary to reproduce the silica‐induced atmospheric drought treatment in other field experiments, we established an outdoor drought experiment in bare‐ground mesocosms at California State University, Los Angeles (CSULA). Our setup combined independent soil drought and atmospheric drought manipulations. This experiment was briefly described in Aguirre et al. (2021). Here, we provide a full examination of the environmental and experimental conditions that influence relative humidity and vapor pressure deficit modification by silica gel packets. Using this design, we explored the following questions:

Q1. How might packet efficacy (i.e., the degree of relative humidity and vapor pressure deficit modification) vary in response to ambient air conditions (relative humidity, temperature, and vapor pressure deficit), soil moisture, and time elapsed since packet deployment?
Q2. Does the degree of air microclimate modification respond in real time to natural diurnal fluctuations in ambient air moisture (relative humidity and vapor pressure deficit)?
Q3. Are there cumulative effects wherein packet efficacy might be dependent on the ambient relative humidity and vapor pressure deficit conditions of the previous day?

## 2 MATERIALS AND METHODS

### 2.1 Study site

This experiment was conducted at CSULA (34.0668° N, 118.1684° W) from January 2 through May 12, 2020, which spans Southern California's Mediterranean growing season. Between 1970 and 2019, this region received an average of 706 mm of precipitation annually (PRISM Climate Group) and 618 mm of rainfall during the typical wet season (November–April). Wet season rainfall varies widely (e.g., from 116 mm in 1977 to 1660 mm in 1973; PRISM Climate Group). During our 2020 growing season, precipitation at CSULA totaled 378 mm and temperature averaged 9.4°C (PRISM Climate Group).

### 2.2 Study design

**FIGURE 1:**
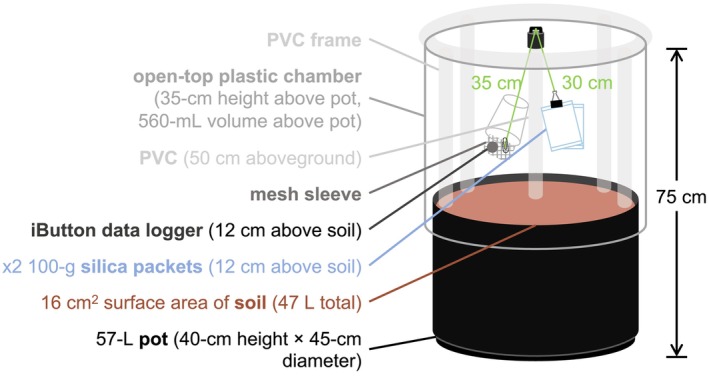
Our experiment featured nine 57‐L pots, each mounted with a 35‐cm‐tall open‐top chamber. We implemented our atmospheric drought treatment by suspending silica gel packets 12 cm above the soil surface and microclimate sensors at the same height.

We filled nine polypropylene planter pots (40‐cm height, 45‐cm diameter, 57‐L volume, CN‐NCL, Greenhouse Megastore) with 47 L of soil obtained nearby from the Stunt Ranch Santa Monica Mountains Reserve in Calabasas, California (34.0939° N, 118.6567° W). Soil at this site has a pH of 7.0 and is composed of 47% sand, 31% silt, and 22% clay (Whelan et al., 2016). To improve drainage, soil was mixed 1:1 with quartzite sand, which was sterilized to avoid the introduction of external microbiota (Aguirre et al., 2021). Around each pot, we mounted open‐top chambers (35‐cm height × 45‐cm diameter) using a PVC frame and 6‐μm‐thick greenhouse film with 92% light transmission (Figure 1; 108,658, Sun Master Pull and Cut Greenhouse Film, Growers Supply, Dyersville, Iowa, USA). This setup allowed for environmental contact from above (via solar irradiance, temperature, relative humidity, and wind flow) while simultaneously providing an enclosure to capture the unique air microclimate within each pot.

Six of the nine total pots represented three true replicates of each of the two experimental drought treatments: (1) atmospheric drought × soil drought and (2) atmospheric drought × ambient soil moisture. To impose atmospheric drought in each of our six experimental pots, we suspended two Dry & Dry 100‐g fabric silica gel packets (Dry & Dry, Brea, California, USA) 12 cm above the soil by a 30‐cm string tied to a 1‐m‐tall PVC tube installed in the center of each pot (Figure 1). We replaced water‐saturated packets with refreshed packets every 0–7 days (see Appendix S1 for our detailed protocol for packet desaturation and reuse). While we did not initially intend to vary the number of days between packet replacements, the COVID‐19 pandemic had a major effect on our ability to access our experiment and facilitated an opportunity to study this variable. As a result, by the end of the study, we had high replication of replacing packets daily (54 instances × 6 pots), every other day (22 instances × 6 pots), and every 2 days (19 instances × 6 pots). We had less replication (≤8 instances) for longer durations of time between replacements.

We maintained soil moisture at either of two watering levels (ambient or drought), which we calculated based on precipitation trends at our site over the past 50 years (PRISM Climate Group). The ambient watering treatment was designed to approximate the overall average of growing season rainfall distributed evenly throughout the season (1630 mL every 4 days; Aguirre et al., 2021). The drought watering treatment fell two standard deviations below that mean (170 mL every 9 days, approximately equal to 4.8 mm annually; Aguirre et al., 2021). Upon observing unexpectedly high mortality of grasses in adjacent pots (which comprised a larger experiment), we adjusted our initial watering regime: all pots received ambient watering for 2 weeks (February 10–21), and beginning February 24, drought pots were watered 170 mL every 4 days (still 10% of the ambient volume) for the remainder of the study (Aguirre et al., 2021). Finally, during one rainy week in March, we installed a large plastic tarp to function as a rainout shelter over the entire experiment (108,658, Sun Master Pull and Cut Greenhouse Film, Growers Supply, Dyersville, IA).

We maintained the remaining pots (*n* = 3) nearby with ambient air humidity × ambient soil moisture and an identical pot and chamber setup as the six experimental pots. We averaged ambient air temperature, relative humidity, and vapor pressure deficit data from these pots (which exhibited nonsignificant between‐pot variability in air microclimate readings) as control reference conditions. We chose to use mesocosm‐scale readings as our control reference conditions as opposed to weather station data because previous studies have shown that small‐scale sensors in control pots located beside experimental pots may serve as a better reference for small‐scale effects (Maclean & Klinges, 2021). However, there are also known issues with using this type of data to make inferences about broader‐scale daytime air conditions (e.g., thermodynamic and evaporative differences directly above soil vs. higher aboveground and in open air; Maclean & Klinges, 2021). Hence, we report comparisons between our mesocosm‐level readings and local weather station data in Figure S1.

### 2.3 Air microclimate monitoring

In all nine pots, we installed one iButton Hygrochron datalogger (DS1923, Maxim Integrated, San Jose, CA) to monitor the air microclimate every 10 min. We enclosed each iButton in a mesh envelope positioned under (but not inside) a white plastic cup that blocked direct irradiance and precipitation (Wright et al., 2015). In each pot, the iButton + cup unit was tied to the central PVC pipe, allowing for the dataloggers to be suspended 12 cm above the soil surface (i.e., at the same height as the silica packets; Figure 1). The iButtons recorded the modified air microclimate (Temp_exp_ and RH_exp_) in the experimental pots and the ambient air microclimate (Temp_amb_ and RH_amb_) in the control reference pots. We used these values to calculate pot vapor pressure deficit (equation in Anderson, 1936), as well (VPD_exp_ and VPD_amb_).

### 2.4 Statistical modeling and analyses

Due to a temporarily malfunctioning datalogger, we excluded one pot (treated with dehumidified air × ambient soil moisture) from our analyses between March 31 and May 12. Data from the other five pots during that window, plus all six pots over the remaining dates, were averaged over hourly, 12‐hour, and 24‐hour intervals.

To represent the difference in relative humidity between a pot with silica packets and a pot of ambient air, we calculated a relative humidity effect (RH_effect_) response variable as.

(1) $${\mathrm{RH}}_{\text{effect}}={\mathrm{RH}}_{\exp}–{\mathrm{RH}}_{\mathrm{amb}}$$

where ${\mathrm{RH}}_{\text{effect}}$ < 0 indicates microclimate dehumidification, ${\mathrm{RH}}_{\text{effect}}$ = 0 indicates no effect of packets on microclimate relative humidity, and ${\mathrm{RH}}_{\text{effect}}$ > 0 indicates microclimate humidification. In the same way, we calculated a vapor pressure deficit effect (VPD_effect_) response variable as:

(2) $${\mathrm{VPD}}_{\text{effect}}={\mathrm{VPD}}_{\exp}–{\mathrm{VPD}}_{\mathrm{amb}}$$

where VPD_effect_ > 0 indicates that packets dehumidified and warmed the microclimate, VPD_effect_ = 0 indicates no effect of packets on microclimate vapor pressure deficit, and VPD_effect_ < 0 indicates that packets humidified and cooled the microclimate.

Using R Statistical Software, we built linear mixed‐effects models using the lmer function from the lmerTest library (Kuznetsova et al., 2017). To investigate how packet modification of relative humidity and vapor pressure deficit might vary in response to ambient air conditions, soil moisture, and time elapsed since packet deployment (Q1), we focused our main analyses on the 12‐hour‐averaged (6:00–18:00) “daytime” effects as these will be most relevant for commonly studied plant behaviors (e.g., photosynthesis during daylight hours). We assigned the daytime RH_effect_ or VPD_effect_ as a continuous response variable and RH_amb_, Temp_amb_, VPD_amb_, watering treatment, count of days since packet replacement, and all higher‐order interactions as fixed effects. We report on the best‐fit model as described below.

To assess whether the degree of air microclimate modification responds in real time to natural diurnal fluctuations in ambient relative humidity and vapor pressure deficit (Q2), we averaged the data by hour. As above, we included the daytime RH_effect_ or VPD_effect_ as a continuous response variable. We included the fixed effects of RH_amb_, Temp_amb_, VPD_amb_, and hour, as well as interactions between hour and any of the three ambient air microclimate predictors. We excluded data recorded beyond 2 days of packet deployment to focus on conditions for which we had higher replication. We report on the best‐fit model as described below.

To test for the presence of cumulative effects wherein packet efficacy might depend on the ambient relative humidity and vapor pressure deficit conditions of the previous day (Q3), we calculated 24‐hour averages of microclimate readings and focused on cumulative 2‐day effects. We expect spillover effects between days to be most straightforward in this format. As above, we assigned the daytime RH_effect_ or VPD_effect_ as a continuous response variable. For predictors, we included the main effect of the previous day's ambient air conditions (${\mathrm{RH}}_{{\mathrm{amb}}_{t-1}}$, ${\text{Temp}}_{{\mathrm{amb}}_{t-1}}$, or ${\mathrm{VPD}}_{{\mathrm{amb}}_{t-1}}$) and its two‐way interaction with the ambient air conditions of the current day, *t* (RH_amb_, Temp_amb_, or VPD_amb_). As above, we excluded all data recorded beyond 2 days of packet deployment. We report on the best‐fit model as described below.

For all models, we included date as a noninteractive fixed effect. This variable was included only to detrend our data and allow us to focus instead on the environmental and experimental influences on packet efficiency. Further, we included pot as a random effect in all models to account for repeated measurements taken in the same pots over time. Finally, we tested three different temporal autocorrelation structures (compound symmetry, first‐order autoregressive, and unstructured) and consistently found that the unstructured format aligned best with our experimental design where the degrees of freedom were closest to the product of pots and dates (Crawley, 2012; Isbell et al., 2015). For example, our main daytime dataset features 571 total rows of data (= (5 pots × 101 dates) + (1 pot × 66 dates)) and our unstructured daytime models both had 548 denominator degrees of freedom.

We performed a model selection analysis on each dataset (daytime, hourly, and 24‐h) and response variable (RH_effect_ and VPD_effect_). We designated the best‐fit models as those with the lowest AIC. We also used df, BIC, and *R* ^2^ to either support these decisions or identify relationships needing further testing. Finally, we analyzed the best‐fit model from each dataset (daytime, hourly, and 24‐h) using the anova function from the stats library (v4.2.2; R Core Team, 2022; RStudio Team, 2021).

## 3 RESULTS

### 3.1 Q1: Daytime effects of ambient air conditions, soil watering treatments, and packet replacement

**FIGURE 2:**
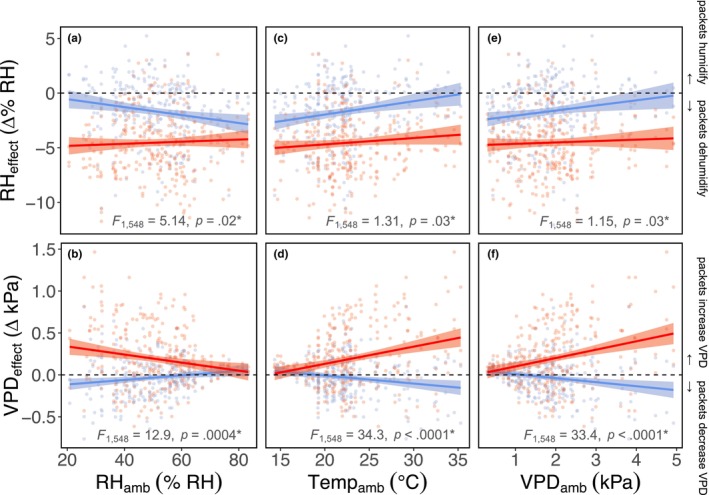
We measured changes in microclimate relative humidity (RH_effect_) and vapor pressure deficit (VPD_effect_) driven by the silica gel packets in comparison with ambient pots nearby. Because vapor pressure deficit is a composite measure of air relative humidity and temperature, we report on the separate roles of ambient relative humidity (a, b) and ambient temperature (c, d), in addition to ambient vapor pressure deficit (e, f). Overall, packets dehumidified and increased vapor pressure deficit more effectively in pots of dry soil (red) than wet soil (blue). Dehumidification effects in wet‐soil pots were stronger on humid days (a), on cool days (c), and when vapor pressure deficit was lower (e). Vapor pressure deficit effects were near zero in wet‐soil pots (b, d, f). But in dry‐soil pots, vapor pressure deficit increases were strongest on dry days (b), on hot days (d), and when vapor pressure deficit was higher (f). Points represent individual dehumidified pots on separate dates, trendlines indicate significant interactions, and bands denote 95% confidence intervals.

**FIGURE 3:**
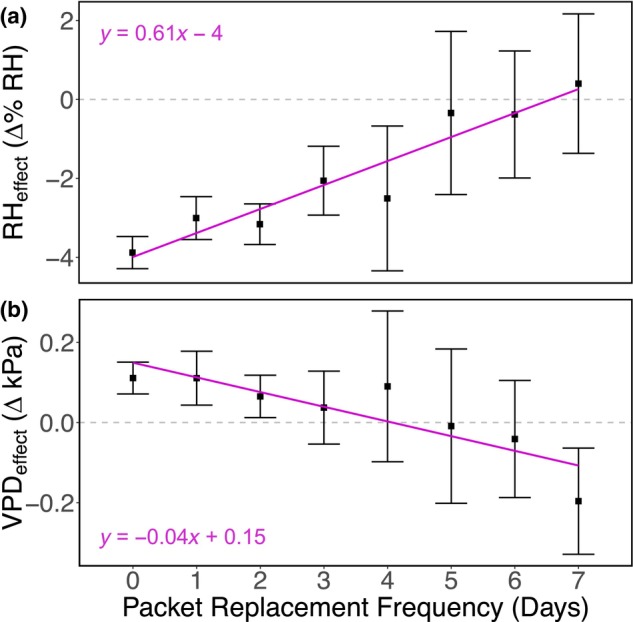
We monitored changes in relative humidity and vapor pressure deficit following packet replacement (on day zero). Packets dehumidified pot microclimates within 4 days of deployment, beyond which dehumidification effects overlapped with zero (a, Days Since Replacement significant main effect; *F*_1,548_ = 31.0, *p* < .0001). Packets increased microclimate vapor pressure deficit within 2 days of deployment, beyond which packet modification of vapor pressure deficit overlapped with zero (b, Days Since Replacement significant main effect; *F*_1,548_ = 6.36, *p* = .01). Trendlines indicate significant relationships and error bars represent 95% confidence intervals around mean points.

We found that the best‐fit model for predicting daytime dehumidification (RH_effect_) by packets included ambient vapor pressure deficit, soil watering regime, days since packet replacement, and all higher order interactions (Table S2). Packet dehumidification was strongest when vapor pressure deficit was low (corresponding with cool, humid conditions; Table 1; Figure 2a,c,e; VPD_amb_ significant main effect; *F*_1,548_ = 13.3, *p* = .0003). Overall, packets dehumidified pot microclimates within 4 days of deployment Table 1; Figure 3a; Days Since Replacement significant main effect; *F*_1,548_ = 31.0, *p* < .0001. We also report evidence that packets dehumidified over longer periods on hot, dry days (Table 1; Figure S2; VPD_amb_ × Days Since Replacement significant interaction; *F*_1,548_ = 7.46, *p* = .01).

**TABLE 1:** Our best‐fit daytime models predicted the RH_effect_ and VPD_effect_ based on VPD_amb_, watering treatment, days since packet replacement, and all higher order interactions.

Predictor	Fixed effects	df	*F*	*p*
RH_effect_	VPD_amb_	1, 548	13.3	.0003*
	Watering	1, 6	4.73	.07
	Days Since Replacement	1, 548	31.0	<.0001*
	Date	1, 549	3.08	.08
	VPD_amb_ × Watering Treatment	1, 548	1.15	.28
	VPD_amb_ × Days Since Replacement	1, 548	7.46	.01*
	Watering Treatment × Days Since Replacement	1, 548	2.19	.14
	VPD_amb_ × Watering Treatment × Days Since Replacement	1, 548	0.50	.48
VPD_effect_	VPD_amb_	1, 548	0.33	.57
	Watering	1, 5	0.17	.70
	Days Since Replacement	1, 548	6.36	.01*
	Date	1, 548	1.22	.27
	VPD_amb_ × Watering Treatment	1, 548	33.4	<.0001*
	VPD_amb_ × Days Since Replacement	1, 548	0.41	.52
	Watering Treatment × Days Since Replacement	1, 548	0.01	.92
	VPD_amb_ × Watering Treatment × Days Since Replacement	1, 548	0.14	.70

*Note*: ANOVA results significant at α = .05 are bolded and asterisked.

In our corresponding analysis of daytime vapor pressure deficit modification by packets (VPD_effect_), we found that the same set of fixed effects (ambient vapor pressure deficit, watering regime, days since packet replacement, and all higher order interactions) produced one of the best‐fit models within five AIC points; we report on this model here in order to make direct comparisons with the analogous relative humidity model (Table S2). Notably, ambient vapor pressure deficit interacted with our watering treatments to jointly influence packet modification of microclimate vapor pressure deficit: on cool, humid days, the VPD_effect_ in all pots overlapped with zero, while on hot, dry days, packets increased vapor pressure deficit in drought‐watered pots but had a near‐zero effect in ambient‐watered pots (Table 1; Figure 2b,d,f; VPD_amb_ × Watering Treatment significant interaction; *F*_1,548_ = 33.4 *p* < .0001). Further, packets increased pot vapor pressure deficit consistently within the first 2 days of deployment (Table 1; Figure 3b; Days Since Replacement significant main effect; *F*_1,548_ = 6.36, *p* = .01).

### 3.2 Q2: Hourly packet effects

**FIGURE 4:**
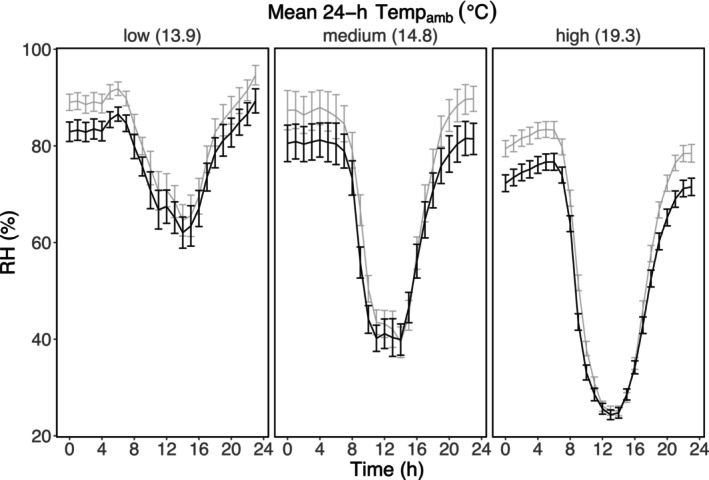
We measured fine‐scale changes in relative humidity over low‐, medium‐, and high‐temperature days. Hourly relative humidity in pots with silica packets (black lines) can be compared with ambient air relative humidity in nearby nontreated pots (gray lines). Both varied with respect to ambient air temperature (panels). Packet dehumidification was stronger, for more hours of the day, on hotter days (Temp_amb_ × Hour significant interaction; *F*_1,11,450_ = 44.7, *p* < .0001). Error bars represent 95% confidence intervals.

The best‐fit model for predicting hourly changes in packet dehumidification included the main effects of ambient air temperature, hour of day, and their interaction term (Table S3). Packets dehumidified pot microclimates during most hours of the day, with the strength of dehumidification shifting continuously in response to diurnal fluctuations in ambient air temperature (Table 2; Figure 4; Temp_amb_ × Hour significant interaction; *F*_1,11,450_ = 44.7, *p* < .0001). Dehumidification ability declined in the morning hours between 8:00 and 10:00 am (Figure 4).

**TABLE 2:** Our best‐fit hourly model predicted the RH_effect_ from the main effects and interaction of Temp_amb_ and hour.

Predictor	Fixed effects	df	*F*	*p*
RH_effect_	Temp_amb_	1, 11,450	50.2	<.0001*
	Hour	1, 11,450	40.1	<.0001*
	Date	1, 11,452	4.07	.04*
	Temp_amb_ × Hour	1, 11,450	44.7	<.0001*

*Note*: ANOVA results significant at α = .05 are bolded and asterisked.

The best‐fit model for predicting hourly changes in packet vapor pressure deficit modification included the main effects of ambient vapor pressure deficit, hour of day, and their interaction term, though because multiple models had similar AIC scores with little improvement in model fit regardless of the variables selected, and all models had low *R* ^2^ values (<.09), we do not report further on those results here (Tables S3 and S4; Figure S3).

### 3.3 Q3: Two‐day cumulative packet effects

We found that the best‐fit model for predicting a two‐day cumulative RH_effect_ included VPD_amb_ and ${\mathrm{VPD}}_{{\mathrm{amb}}_{t-1}}$ (Table S5) whose interaction was nonsignificant (Table S6; ${\mathrm{VPD}}_{{\mathrm{amb}}_{t-1}}$ × VPD_amb_ nonsignificant interaction; *F*_1,206_ = 1.70, *p* = .19). The best‐fit model for a corresponding 2‐day cumulative VPD_effect_ included the same predictors (VPD_amb_ and ${\mathrm{VPD}}_{{\mathrm{amb}}_{t-1}}$, Table S5) whose interaction was again nonsignificant (Table S6; ${\mathrm{VPD}}_{{\mathrm{amb}}_{t-1}}$ × VPD_amb_ nonsignificant interaction; *F*_1,206_ = 2.48, *p* = .12).

## 4 DISCUSSION

In our outdoor mesocosm experiment, we found that silica packets dried air microclimates by decreasing relative humidity and increasing vapor pressure deficit most effectively when soil moisture was low. The 5% RH reduction capacity we observed was sufficient to increase vapor pressure deficit by up to 0.4 kPa (a value similar to 50‐year projections for vapor pressure deficit, Ficklin & Novick, 2017). We also found that packets dehumidified most reliably when replaced prior to their saturation with captured moisture (see Appendix S1 for our protocol for desaturating used packets for redeployment). As such, replacing packets within 2 days helped to maintain drought‐level vapor pressure deficit at our experimental site in Los Angeles, CA. We found contrasting effects of ambient vapor pressure deficit on packet modification of microclimate relative humidity versus microclimate vapor pressure deficit, indicating areas for future experimentation that we explore below. We emphasize that there are tradeoffs in this approach: packets likely cannot be deployed at very large scales or at remote sites that are challenging to access regularly. As such, future work should examine high‐precision, automated feedback systems for maintaining outdoor relative humidity and vapor pressure deficit at optimum ranges in such scenarios (as suggested in Wright & Collins, 2023).

### 4.1 Packet performance relative to soil moisture and air conditions

In our arid Mediterranean climate, we found that packets reduced ambient air relative humidity by 2%–3% in pots of wet soil when ambient vapor pressure deficit was lower than 2.5 kPa. Because reporting on limits for vapor pressure deficit is not immediately intuitive for applying these results experimentally (e.g., weather forecasts usually do not include vapor pressure deficit forecasts), we thus report on temperature and relative humidity optima, as well: packets dehumidified pots of wet soil when pot relative humidity was above 45% and ambient pot temperatures were below 25°C. These microclimate readings corresponded with weather station readings of 33.5% RH and 33.5°C (Figure S1). But when packets were combined with soil drought, they reduced pot relative humidity by 5% regardless of ambient air temperature, relative humidity, or vapor pressure deficit. Similarly, packets increased pot vapor pressure deficit more reliably in drought‐watered pots than ambient‐watered pots; this VPD_effect_ was strongest (up to 0.4 kPa) on days of high vapor pressure deficit (and thus high atmospheric temperatures and low relative humidity). This evidence suggests that packet efficacy is related to cycling of water through multiple water storage pools in these mesocosms (e.g., Figure S4). While packets can be used to remove water from the air, this air moisture might be rapidly replaced by evaporated soil surface water if soil moisture is high (Figure S4b). But in drier soils, soil moisture may not be high enough to drive evaporation into dry air, which would allow us to detect stronger reductions in atmospheric humidity in dry pots (Figure S4a).

Importantly, we also present evidence of an apparent opposition between how silica packets modify microclimate relative humidity versus vapor pressure deficit under the context of shifting ambient vapor pressure deficit: daytime increases in ambient vapor pressure deficit corresponded with a stronger VPD_effect_ but a weaker RH_effect_. We speculate this trend to be the result of packets' different physical responses to changes in humidity and temperature. In particular, packets may dehumidify more effectively with increasing ambient humidity due to passive concentration gradients: higher humidity may cause greater diffusion of water molecules toward the silica desiccant. At the same time, packets may capture moisture more effectively at higher temperatures when rates of water transfer increase. Warming drives higher rates of evaporation and soil absorption (Dai et al., 2004; Dirmeyer et al., 2012; Hanks, 2012; Huntington, 2006; Samaniego et al., 2018; Smith et al., 2008; Walker, 1994). We thus present some clues that packets may dehumidify best when ambient air is hot and humid, though this was impossible to test directly in our arid Mediterranean climate where all of the hottest days were also the driest days. To address this gap, we recommend that future studies apply desiccation packets in tropical wet systems.

The vapor pressure deficit modification we report here (+0.4 kPa) corresponds with meaningful ecological differences. For example, Ficklin and Novick (2017) report on vapor pressure deficit differences of approximately 0.5 kPa between central Minnesota and arid regions of Kansas. Past work has emphasized the role that relative humidity and vapor pressure deficit changes at these magnitudes can play for plant performance. For example, Grossiord et al. (2020) report that vapor pressure deficit changes ≥0.1 kPa can decrease steady‐state stomatal aperture, stomatal conductance, and CO_2_ assimilation rate. Another study by Schönbeck et al. (2022) found that vapor pressure deficit levels as low as 1.4 kPa can lead to losses of stem conductivity, leaf water potential, and biomass, even when soil water is not limiting.

### 4.2 Packets as rapid feedback systems

We found that packets can be sensitive to both immediate and cumulative changes in air conditions. For example, in our data, packets dehumidified pot microclimates from 6:00 pm to 6:00 am (overnight, when humidity was most abundant), emitted humidity around 8:00 am (possibly from oversaturation with nighttime humidity), and then returned to dehumidification at 9:00 am. This switch may have been driven by increases in water transfer rates at sunrise when the air warmed. Simultaneously, daytime warming likely expedited the evaporation of soil surface moisture, creating increased humidity in pots of wet soils especially. As such, mornings at our site are correlated with reduced efficiency of relative humidity effects but higher efficiency of vapor pressure deficit effects.

Past plant physiological studies have shown that stomatal pore aperture and gas exchange operate at very fine‐scale time intervals (Grossiord et al., 2020; McAdam & Brodribb, 2015). For example, plants reduce leaf water potential, stem hydraulic conductance, and stomatal pore aperture in response to soil moisture deficits within minutes (Christmann et al., 2007; Lawson & Blatt, 2014; Saliendra et al., 1995). Understanding fast‐acting physiological responses like these under the context of drought requires the implementation of a rapid‐response drought manipulation. Our results suggest that silica packets can perform microclimate modification in real time.

### 4.3 Packet dehumidification on cumulative days following deployment

In our assessment of cumulative 2‐day packet effects, we found no evidence that the previous day's ambient air vapor pressure deficit impacted packet modification of vapor pressure deficit on the present day. This aligns with our finding that packets can reduce air humidity to atmospheric drought levels within the first 3 days of deployment. Taken together, both results suggest that packets can be deployed for consecutive days and still retain their capacity to reduce atmospheric humidity and increase vapor pressure deficit.

### 4.4 Study limitations

Despite low replication of experimental units (3 pots per soil moisture level), we found robust and consistent effects of the desiccation packets. Our ability to detect these effects, despite a small number of pots, may partly be due to the high temporal resolution of humidity and temperature data collected in our pots (measurements every 10 min), which is higher resolution relative to other drought experiments (e.g., Adair et al., 2011; Fischer et al., 2019; Leimer et al., 2014; Vogel et al., 2013; Wang et al., 2016; Wright et al., 2015). Due to budget and time constraints, there is often a tradeoff between measuring soil moisture either more frequently across a smaller number of experimental units (as in our study) or less frequently across a larger number of experimental units (as in many other drought studies). Here, we find overwhelmingly consistent effects over time. We encourage future studies to implement higher replication, which could increase the precision of these estimated treatment effects. However, we do not expect that increasing replication would change the direction or significance of these effects.

While this experiment was performed in bare‐ground mesocosms, future studies that seek to implement an atmospheric drought treatment using silica gel packets, especially when vegetation is present, should consider the challenges of scaling up this approach to field experiments. For example, in the context of large, open‐field plots (in comparison to mesocosms), there may be greater mixing of air across plot edges. Specifically, if the air on one side of a plot edge becomes substantially more humid (due to treatment effects, landscape effects, or natural environmental fluctuations), the humidity may diffuse and equilibrate beyond the plot margin. Furthermore, it may be necessary to modify the amount of desiccant applied relative to the size of the plot and the amount of airflow impeded by infrastructure (rainout shelters, mesocosm walls, etc.).

Better understanding of the full mesocosm water cycle will also help apply these results more broadly (e.g., Figure S4). Further, packet performance will likely differ when vegetation is present as plants connect soil moisture to air humidity through feedbacks in both transpiration and shading, which can affect microclimate temperature and rates of evaporation (Wright et al., 2014). Finally, tracking how silica dehumidification (or industrial forms of dehumidification) may drive increased rates of soil moisture loss will be an important next step in the design of atmospheric drying experiments.

### 4.5 Simulating future atmospheric drying

Atmospheric drought will become more common and more severe as climate change continues to occur. Overlooking the impacts of atmospheric drying during drought manipulations can underestimate the biological mechanisms that drive drought resistance in plant communities (Aguirre et al., 2021; Grossiord et al., 2020; Huynh et al., 2023; Ocheltree et al., 2014; Watson et al., 2023). To advance our understanding of how drought events will impact plant communities, we must conduct drought experiments with both soil and air drying regimes that accurately simulate natural ecological drought, which is characterized by moisture shortages at both the soil and atmospheric levels (Aguirre et al., 2021; IPCC, 2021; Novick et al., 2016; Wright & Collins, 2023). This study examined the efficacy of silica gel packets as one such dehumidification solution.

## AUTHOR CONTRIBUTIONS

**S. Varghese:** Conceptualization (supporting); data curation (lead); formal analysis (lead); funding acquisition (supporting); investigation (lead); methodology (supporting); project administration (lead); visualization (lead); writing – original draft (lead); writing – review and editing (lead). **B. A. Aguirre:** Conceptualization (lead); data curation (supporting); funding acquisition (supporting); investigation (supporting); methodology (supporting); writing – original draft (supporting); writing – review and editing (supporting). **F. Isbell:** Formal analysis (supporting); investigation (supporting); writing – original draft (supporting); writing – review and editing (supporting). **A. J. Wright:** Conceptualization (lead); data curation (supporting); formal analysis (supporting); funding acquisition (lead); investigation (supporting); methodology (lead); project administration (supporting); resources (lead); visualization (supporting); writing – original draft (supporting); writing – review and editing (supporting).

## CONFLICT OF INTEREST STATEMENT

The authors declare no conflicts of interest.

### OPEN RESEARCH BADGES

This article has earned an Open Data badge for making publicly available the digitally‐shareable data necessary to reproduce the reported results. The data is available at https://doi.org/10.6084/m9.figshare.19611819.v1; https://doi.org/10.6084/m9.figshare.19611822.v1; https://doi.org/10.6084/m9.figshare.19611855.v1; https://doi.org/10.6084/m9.figshare.19611813.v1; https://doi.org/10.6084/m9.figshare.19611816.v1 (Varghese et al., 2022a, 2022b, 2022c, 2022d, 2022e).

## Supporting information


Appendix S1

## Data Availability

Data are organized into five separate spreadsheets publicly available at https://figshare.com/projects/Simulating_atmospheric_drought_Silica_gel_packets_effectively_dehumidify_microclimates/137385.
