# Supplementary material for: Simulating atmospheric drought: Silica gel packets dehumidify mesocosm microclimates
Source: Ecol Evol. 2024 Aug 21;14(8):e70139. doi: 10.1002/ece3.70139 (PMC11336202; doi:10.1002/ece3.70139)
Supplement: Supplementary file 1 — Appendix S1 [file ECE3-14-e70139-s001.pdf]

Supporting Information

Title: Simulating atmospheric drought: Silica gel packets dehumidify mesocosm microclimates

Running Head: Simulating atmospheric drought with silica packets

Varghese, S.[1,2]*, Aguirre, B.A.[3], Isbell, F.[2], Wright, A.J.[1]

[1] California State University Los Angeles, Department of Biological Sciences, Los Angeles, CA

[2] University of Minnesota, Department of Ecology, Evolution, and Behavior, Minneapolis, MN

[3] Cornell University, Department of Ecology and Evolutionary Biology, Ithaca, NY

Corresponding Author

*Steph Varghese

steph-v@umn.edu

140 Gortner Laboratory, 1479 Gortner Avenue, St. Paul, MN 55108

Co-Authors

Beatriz A. Aguirre baa84@cornell.edu

Forest Isbell isbell@umn.edu

Alexandra J. Wright awrigh20@calstatela.edu

Table S1. Defining different types of drought according to the IPCC 2021 report (supported in Van Loon, 2015; Crausbay et al., 2017; Wright & Collins, 2023).

**Drought Term**	**Definition**	**Real-World Impacts**
meteorological drought	a shortage of precipitation	reduced soil moisture; hydrological drought, agricultural drought, and socioeconomic drought
hydrological drought	a deficit in streamflow	below-normal levels in groundwater, lakes, and reservoirs; declining wetland area; decreased river discharge
agricultural drought	the result of agricultural systems experiencing a combination of precipitation deficits and increased evaporative demand, which interactively reduce soil moisture and ultimately inhibit crop production	reduced crop quality or yield
ecological drought	the result of ecological systems experiencing a combination of precipitation deficits and increased evaporative demand, which interactively reduce soil moisture and ultimately limit ecosystem function	ecosystems are driven beyond thresholds of vulnerability, resulting in cascading negative impacts on ecosystem services

Table S2. Model selection results for our daytime dataset. All models included Date as a non-interactive fixed effect and a random effect of Pot. The best fit model is indicated with an asterisk.

Response	Predictors	df	AIC	BIC	conditional R^2
RH_{effect}	RH_{amb}	5	2734	2756	0.439
	$Temp_{amb}$	5	2733	2755	0.439
	VPD_{amb}	5	2731	2753	0.437
	Watering Treatment	5	2722	2744	0.423
	Days Since Replacement	5	2636	2658	0.482
	RH_{amb} × $Temp_{amb}$	7	2753	2784	0.438
	RH_{amb} × VPD_{amb}	7	2743	2774	0.440
	RH_{amb} × Watering Treatment	7	2729	2759	0.433
	RH_{amb} × Days Since Replacement	7	2645	2675	0.491
	$Temp_{amb}$ × VPD_{amb}	7	2741	2771	0.444
	$Temp_{amb}$ × Watering Treatment	7	2728	2759	0.437
	$Temp_{amb}$ × Days Since Replacement	7	2631	2661	0.500
	VPD_{amb} × Watering Treatment	7	2725	2755	0.430
	VPD_{amb} × Days Since Replacement	7	2633	2663	0.491
	Watering Treatment × Days Since Replacement	7	2630	2661	0.474
	RH_{amb} × $Temp_{amb}$ × VPD_{amb}	11	2746	2794	0.475
	RH_{amb} × $Temp_{amb}$ × Watering Treatment	11	2765	2813	0.434
	RH_{amb} × $Temp_{amb}$ × Days Since Replacement	11	2675	2722	0.499
	RH_{amb} × VPD_{amb} × Watering Treatment	11	2749	2796	0.432
	RH_{amb} × VPD_{amb} × Days Since Replacement	11	2666	2713	0.493
	RH_{amb} × Watering Treatment × Days Since Replacement	11	2649	2697	0.489
	$Temp_{amb}$ × VPD_{amb} × Watering Treatment	11	2743	2791	0.441
	$Temp_{amb}$ × VPD_{amb} × Days Since Replacement	11	2651	2699	0.501
	$Temp_{amb}$ × Watering Treatment × Days Since Replacement	11	2629	2677	0.506
	VPD_{amb} × Watering Treatment × Days Since Replacement	11	2628*	2676	0.491
	RH_{amb} × $Temp_{amb}$ × VPD_{amb} × Watering Treatment	19	2767	2849	0.488
	RH_{amb} × $Temp_{amb}$ × VPD_{amb} × Days Since Replacement	19	2707	2789	0.522
	RH_{amb} × $Temp_{amb}$ × Watering Treatment × Days Since Replacement	19	2709	2792	0.504
	RH_{amb} × VPD_{amb} × Watering Treatment × Days Since Replacement	19	2687	2769	0.495
	$Temp_{amb}$ × VPD_{amb} × Watering Treatment × Days Since Replacement	19	2665	2747	0.508
	RH_{amb} × $Temp_{amb}$ × VPD_{amb} × Watering Treatment × Days Since Replacement	35	2772	2924	0.540
VPD_{effect}	RH_{amb}	5	112	133	0.432

Temp$_{amb}$	5	104	126	0.435
VPD$_{amb}$	5	101	123	0.436
Watering Treatment	5	102	124	0.454
Days Since Replacement	5	77	99	0.457
RH$_{amb}$ × Temp$_{amb}$	7	134	164	0.435
RH$_{amb}$ × VPD$_{amb}$	7	124	154	0.439
RH$_{amb}$ × Watering Treatment	7	104	135	0.476
RH$_{amb}$ × Days Since Replacement	7	105	136	0.458
Temp$_{amb}$ × VPD$_{amb}$	7	123	153	0.435
Temp$_{amb}$ × Watering Treatment	7	59	90	0.527
Temp$_{amb}$ × Days Since Replacement	7	95	125	0.462
VPD$_{amb}$ × Watering Treatment	7	57	88	0.518
VPD$_{amb}$ × Days Since Replacement	7	91	121	0.461
Watering Treatment × Days Since Replacement	7	86	117	0.482
RH$_{amb}$ × Temp$_{amb}$ × VPD$_{amb}$	11	161	209	0.452
RH$_{amb}$ × Temp$_{amb}$ × Watering Treatment	11	117	165	0.527
RH$_{amb}$ × Temp$_{amb}$ × Days Since Replacement	11	153	201	0.463
RH$_{amb}$ × VPD$_{amb}$ × Watering Treatment	11	102	150	0.526
RH$_{amb}$ × VPD$_{amb}$ × Days Since Replacement	11	140	188	0.464
RH$_{amb}$ × Watering Treatment × Days Since Replacement	11	119	166	0.502
Temp$_{amb}$ × VPD$_{amb}$ × Watering Treatment	11	94	142	0.528
Temp$_{amb}$ × VPD$_{amb}$ × Days Since Replacement	11	129	177	0.464
Temp$_{amb}$ × Watering Treatment × Days Since Replacement	11	62	109	0.556
VPD$_{amb}$ × Watering Treatment × Days Since Replacement	11	58	105	0.544
RH$_{amb}$ × Temp$_{amb}$ × VPD$_{amb}$ × Watering Treatment	19	170	252	0.555
RH$_{amb}$ × Temp$_{amb}$ × VPD$_{amb}$ × Days Since Replacement	19	233	316	0.472
RH$_{amb}$ × Temp$_{amb}$ × Watering Treatment × Days Since Replacement	19	176	258	0.560
RH$_{amb}$ × VPD$_{amb}$ × Watering Treatment × Days Since Replacement	19	153	235	0.554
Temp$_{amb}$ × VPD$_{amb}$ × Watering Treatment × Days Since Replacement	19	127	210	0.561
RH$_{amb}$ × Temp$_{amb}$ × VPD$_{amb}$ × Watering Treatment × Days Since Replacement	35	326	477	0.577

Table S3. Model selection results for our hourly dataset. All models included Date as a non-interactive fixed effect and a random effect of Pot. The best fit model is indicated with an asterisk.

**Response**	**Predictors**	**df**	**AIC**	**BIC**	**conditional R^2**
RH_{effect}	Hour	5	70742	70779	0.178
	RH_{amb} + Hour	6	69007	69051	0.292
	$Temp_{amb}$ + Hour	6	68932	68976	0.300
	VPD_{amb} + Hour	6	69165	69209	0.283
	RH_{amb} × Hour	7	68971	69023	0.296
	$Temp_{amb}$ × Hour	7	68900*	68952	0.303
	VPD_{amb} × Hour	7	69170	69221	0.284
VPD_{effect}	Hour	5	15856	15893	0.081
	RH_{amb} + Hour	6	15869	15913	0.082
	$Temp_{amb}$ + Hour	6	15855	15899	0.083
	VPD_{amb} + Hour	6	15856	15900	0.082
	RH_{amb} × Hour	7	15856	15907	0.084
	$Temp_{amb}$ × Hour	7	15867	15918	0.083
	VPD_{amb} × Hour	7	15865	15917	0.083

Table S4. Our best-fit hourly model predicted the VPD_{effect} from the main effects and interaction of RH_{amb} and hour. ANOVA results significant at $\alpha = 0.05$ are bolded and asterisked.

**Predictor**	**Fixed effects**	**df**	**F**	**p**
VPD_{effect}	RH_{amb}	1, 11450	21.4	**<0.0001***
	Hour	1, 11450	34.1	**<0.0001***
	Date	1, 11454	6.07	**0.01***
	RH_{amb} × Hour	1, 11450	34.3	**<0.0001***

Table S5. Model selection results for our 24-hour dataset. All models included Date as a non-interactive fixed effect and a random effect of Pot. For each response variable, the best fit model involving an interaction is indicated with an asterisk.

Response	Predictors	df	AIC	BIC	conditional R^2
RH_{effect}	$RH_{amb_{t-1}}$	5	987	1003	0.434
	$Temp_{amb_{t-1}}$	5	981	998	0.436
	$VPD_{amb_{t-1}}$	5	976	993	0.445
	$RH_{amb} \times RH_{amb_{t-1}}$	7	1007	1031	0.434
	$RH_{amb} \times Temp_{amb_{t-1}}$	7	997	1020	0.445
	$RH_{amb} \times VPD_{amb_{t-1}}$	7	990	1014	0.446
	$Temp_{amb} \times Temp_{amb_{t-1}}$	7	989	1012	0.456
	$Temp_{amb} \times VPD_{amb_{t-1}}$	7	984	1007	0.455
	$VPD_{amb} \times VPD_{amb_{t-1}}$	7	979*	1003	0.446
VPD_{effect}	$RH_{amb_{t-1}}$	5	-154	-138	0.402
	$Temp_{amb_{t-1}}$	5	-174	-157	0.442
	$VPD_{amb_{t-1}}$	5	-172	-155	0.432
	$RH_{amb} \times RH_{amb_{t-1}}$	7	-128	-104	0.415
	$RH_{amb} \times Temp_{amb_{t-1}}$	7	-147	-123	0.450
	$RH_{amb} \times VPD_{amb_{t-1}}$	7	-148	-124	0.437
	$Temp_{amb} \times Temp_{amb_{t-1}}$	7	-152	-128	0.450
	$Temp_{amb} \times VPD_{amb_{t-1}}$	7	-154	-130	0.439
	$VPD_{amb} \times VPD_{amb_{t-1}}$	7	-159*	-135	0.437

Table S6. Our best-fit 24-hour model predicted the RH_{effect} and VPD_{effect} from the main effects and interaction of VPD_{amb} and $VPD_{amb_{t-1}}$. ANOVA results significant at $\alpha = 0.05$ are bolded and asterisked.

**Predictor**	**Fixed effects**	**df**	**F**	**p**
RH_{effect}	VPD_{amb}	1, 206	1.28	0.26
	$VPD_{amb_{t-1}}$	1, 206	0.14	0.71
	Date	1, 207	6.27	**0.01***
	$VPD_{amb} \times VPD_{amb_{t-1}}$	1, 206	1.70	0.19
VPD_{effect}	VPD_{amb}	1, 206	0.75	0.39
	$VPD_{amb_{t-1}}$	1, 206	0.23	0.64
	Date	1, 207	1.31	0.25
	$VPD_{amb} \times VPD_{amb_{t-1}}$	1, 206	2.48	0.12

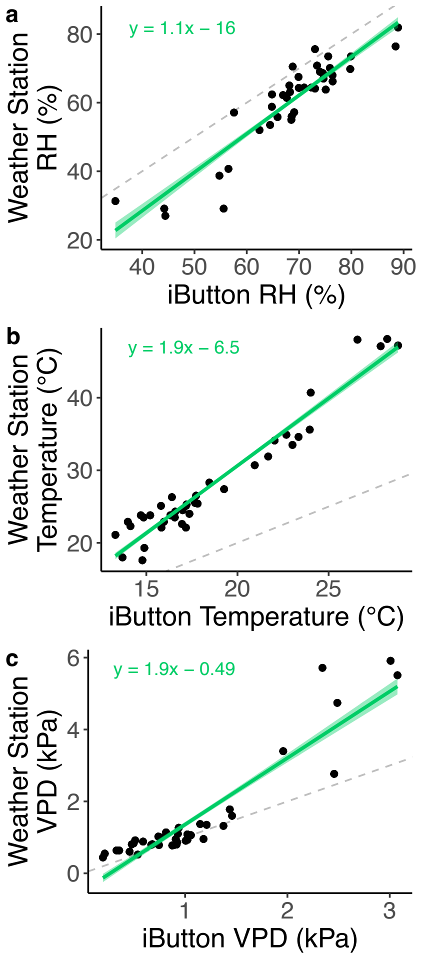

Figure S1. We compare pot microclimate factors (relative humidity, temperature, and vapor pressure deficit) to weather station data. Although small-scale sensors in control pots located beside experimental pots may serve as a better reference for mesocosm effects, there are known issues with using this type of data to make inferences about daytime air conditions (MacLean et al., 2021). Hence, we report relative humidity (a), temperature (b), and vapor pressure deficit (c) comparisons between our mesocosm-level readings (measured at CSULA, located 34.0668 °N and 118.1684 °W) and local weather station data (measured at Bob Hope Airport Weather Station, located 34.20045 °N and 118.35873 °W). Dashed lines represent perfect 1:1 correlation, green trendlines indicate significant relationships, and green bands denote 95% confidence intervals.

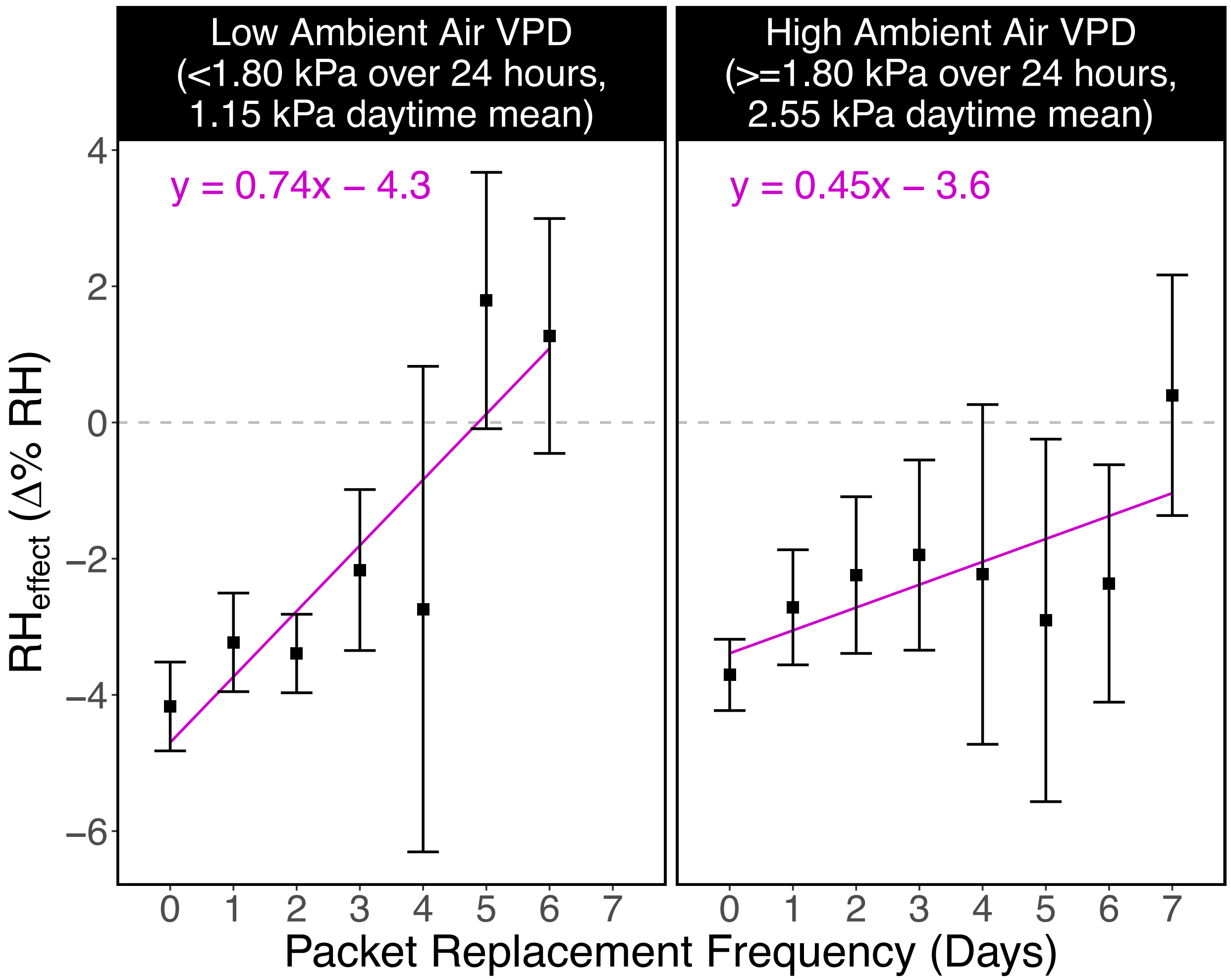

Figure S2. We measured changes in relative humidity as a function of packet replacement frequency and average daily vapor pressure deficit. Packets retained longer-term dehumidification capacity (and thus required less frequent replacement) on hot, dry days (of high ambient vapor pressure deficit). Trendlines indicate significant relationships and error bars represent 95% confidence intervals around mean points.

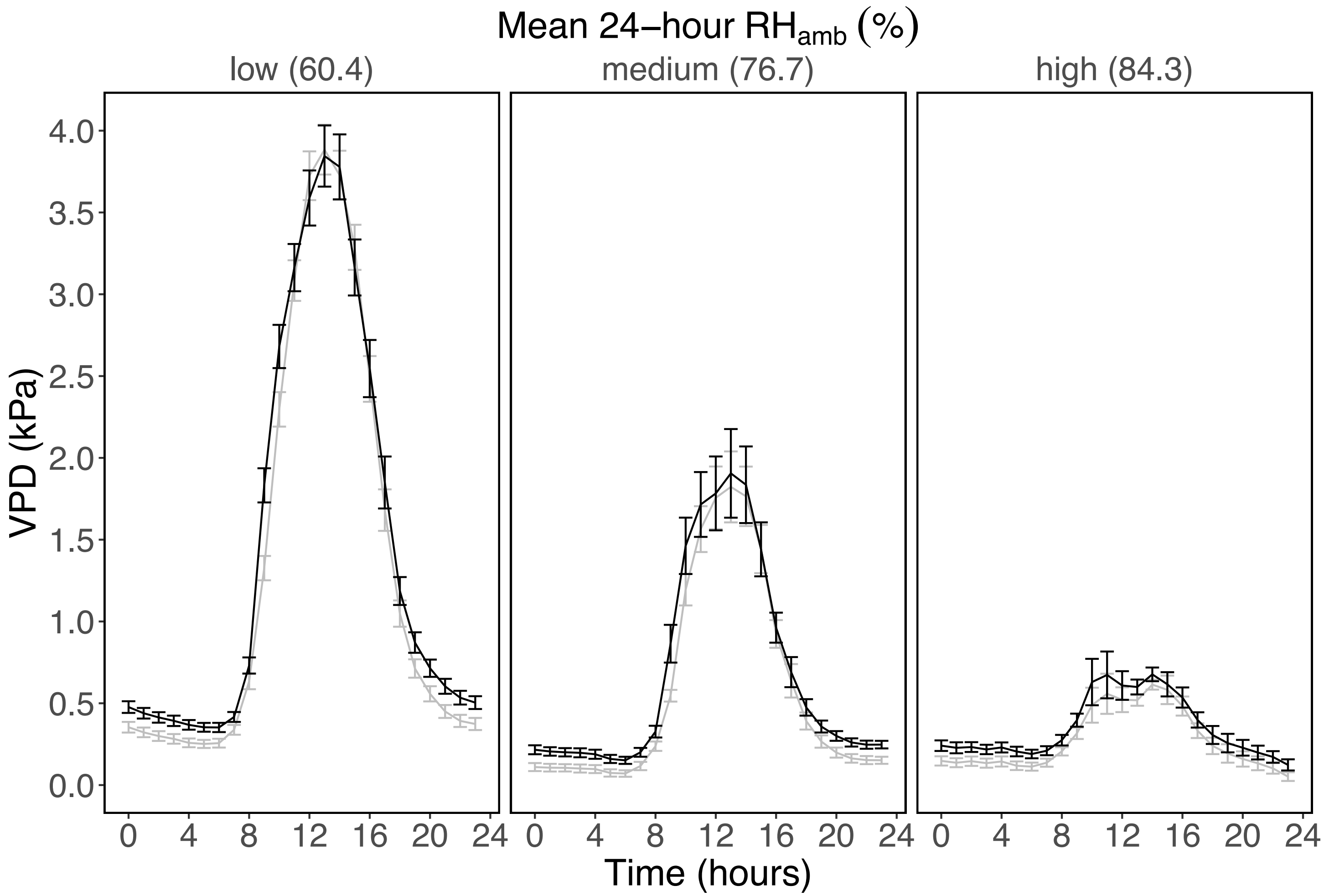

Figure S3. We measured fine-scale changes in vapor pressure deficit over the course of a day. Hourly vapor pressure deficit in pots with silica packets (black lines) can be compared with ambient air vapor pressure deficit in nearby non-treated pots (gray lines). Both varied with respect to ambient air relative humidity (panels). Error bars represent 95% confidence intervals.

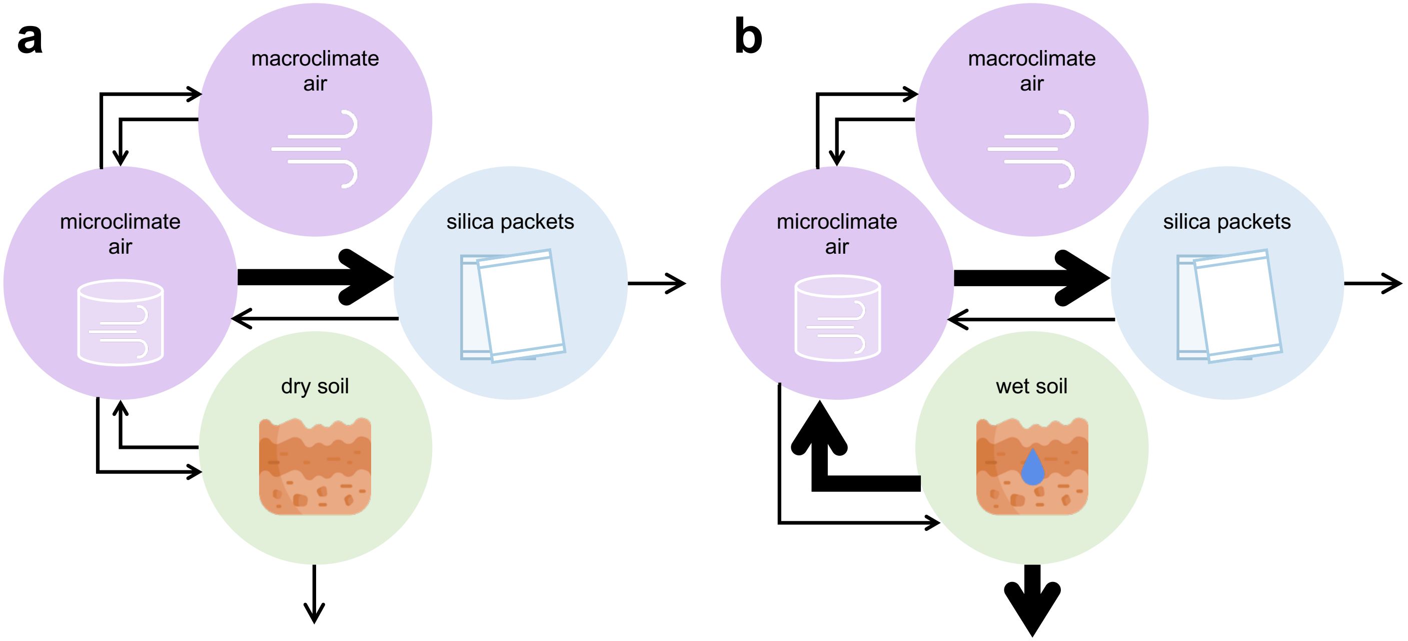

Figure S4. In this conceptualization, the movement of water through a miniature water cycle captured by our mesocosms is indicated via arrows between pools. Due to our open-top chamber design, water vapor is exchanged between the macroclimate and microclimate. Water is also absorbed by the soil and evaporates off the surface of the soil. Silica packets can either capture microclimate humidity or re-emit it, depending on water concentration gradients. In this way, soil moisture can drive packet efficiency. In pots of wet soil (**b**), even if packets are capturing humidity, rapid soil moisture evaporation can replace it. In pots of dry soil (**a**), there is less soil moisture evaporation, and thus packets can maintain microclimate humidity at lower levels. Designed using images from Flaticon.com.

Protocol for desaturating used packets for redeployment.

Silica gel packets can be reused repeatedly by drying out the humidity they captured in the field. In our study, after removing saturated packets from our mesocosms, we transferred them to a Fisher Scientific drying oven for approximately 2-3 days. To drive off any remaining moisture, we heated 8-10 packets at a time in a 1250-watt microwave oven (NN-SN966S, Panasonic) on a defrost setting for 15-20 minutes. Ideal dry mass was ≤107.0 g (as determined by warming 10 packets until they stopped losing moisture, and then averaging their individual weights). Once saturated packets were dried to ≤107.0 g, we immediately stored them in airtight plastic bags until redeployment.
